# Novel Analysis Identifying Functional Connectivity Patterns Associated with Posttraumatic Stress Disorder

**DOI:** 10.1177/24705470221092428

**Published:** 2022-04-18

**Authors:** Natalie Wright,, Ronak Patel,, Sarah J. Chaulk,, Gillian Alcolado,, David Podnar,, Natalie Mota,, Candice M. Monson,, Todd A. Girard,, Ji Hyun Ko,

**Affiliations:** 1 Department of Human Anatomy and Cell Science, Max Rady College of Medicine, 423134 Rady Faculty of Health Sciences, University of Manitoba, Winnipeg, MB, Canada; 2 Neuroscience Research Program, Kleysen Institute for Advanced Medicine, Winnipeg, MB, Canada; 3 Department of Clinical Health Psychology, Max Rady College of Medicine, 423134 Rady Faculty of Health Sciences, University of Manitoba, Winnipeg, MB, Canada; 4 Department of Psychology, 7984 Ryerson University, Toronto, ON, Canada

**Keywords:** resting-state fMRI, PTSD, connectivity, biomarker, graph theory, principal component analysis

## Abstract

Posttraumatic stress disorder (PTSD) is a prevalent psychiatric disorder that can result from experiencing traumatic events. Accurate diagnosis and optimal treatment strategies can be difficult to achieve, due to the heterogeneous etiology and symptomology of PTSD, and overlap with other psychiatric disorders. Advancing our understanding of PTSD pathophysiology is therefore critical. While functional connectivity alterations have shown promise for elucidating the neurobiological mechanisms of PTSD, previous findings have been inconsistent. Eleven patients with PTSD in our first cohort (PTSD-A) and 11 trauma-exposed controls (TEC) underwent functional magnetic resonance imaging. First, we investigated the intrinsic connectivity within known resting state networks (eg, default mode, salience, and central executive networks) previously implicated in functional abnormalities with PTSD symptoms. Second, the overall topology of network structure was compared between PTSD-A and TEC using graph theory. Finally, we used a novel combination of graph theory analysis and scaled subprofile modeling (SSM) to identify a disease-related, covarying pattern of brain network organization. No significant group differences were found in intrinsic connectivity of known resting state networks and graph theory metrics (clustering coefficients, characteristic path length, smallworldness, global and local efficiencies, and degree centrality). The graph theory/SSM analysis revealed a topographical pattern of altered degree centrality differentiating PTSD-A from TEC. This PTSD-related network pattern expression was additionally investigated in a separate cohort of 33 subjects who were scanned with a different MRI scanner (22 patients with PTSD or PTSD-B, and 11 healthy trauma-naïve controls or TNC). Across all participant groups, pattern expression scores were significantly lower in the TEC group, while PTSD-A, PTSD-B, and TNC subject profiles did not differ from each other. Expression level of the pattern was correlated with symptom severity in the PTSD-B group. This method offers potential in developing objective biomarkers associated with PTSD. Possible interpretations and clinical implications will be discussed.

## Introduction

Posttraumatic stress disorder (PTSD) is a psychiatric disorder that interferes significantly with social and occupational functioning.^1^ PTSD can arise when individuals experience or observe traumatic events, such as witnessing actual or threatened death, serious injury, or sexual violation. According to the Diagnostic and Statistical Manual of Mental Disorders (DSM-5),^1^ PTSD symptoms are categorized into four clusters: intrusion, avoidance, negative cognition and mood, and arousal and reactivity. Some common symptoms include flashbacks, disturbances in sleep and concentration, and cued psychological and physiological distress. The lifetime prevalence of PTSD in Canada is estimated to be 9.2%, with current PTSD (symptoms present/lasting for at least 1 month) prevalence estimated to be 2.4%.^2^

Although significant advances have been made towards treating PTSD,^3^ a sizable proportion of individuals either do not respond to available treatments (ie, psychological interventions or psychopharmacology), or only experience reduced severity, rather than full recovery.^4,5^ The heterogeneous etiology and symptom expression of PTSD, and its overlap with other mental health conditions, can make accurate diagnosis and determining optimal treatment strategies difficult. Garnering a reliable way to identify and monitor PTSD using non-invasive procedures such as functional magnetic resonance imaging (fMRI) would be useful in clinical and disability contexts.^6^

Recent advances in fMRI analytic methodology have allowed for a more comprehensive assessment of brain network interactions in neuroimaging research. According to the neurovascular coupling hypothesis, heightened neuronal activity increases regional cerebral blood flow, subsequently increasing the fMRI signal known as blood oxygen level dependency (BOLD.^7^^)^ The neurovascular coupling hypothesis posits that synchronously fluctuating fMRI signals from two distinct brain regions indicate that those two regions are functionally communicating.^8^ Focus has shifted from isolated regional activation toward atypical synchronous neural interactions found in psychiatric disorders, and suggested that disconnection or hyper-connection between brain regions can be more relevant to clinical symptom expression than regional dysfunction.^9^

The triple network model^9^ posits that disturbances in connectivity of three key networks play significant roles in many psychiatric disorders, including PTSD. These networks – the Salience Network (SN), Default Mode Network (DMN), and Central Executive Network (CEN) – are associated with processes impaired in PTSD, such as extinction learning,^10^ autonomic and emotional regulation, conflict monitoring, and reward processing.^11^ Increased connectivity was found in SN^12^ and CEN while mixed results were associated with DMN (increased^12^ vs. decreased.^13,14^^)^ It should be noted that other brain regions outside of those core triple networks, such as the precentral gyri, supramarginal gyrus, and superior parietal gyrus, also displayed significantly increased connectivity.^12^ Thus, while investigating these three networks has shed light on connectivity alterations in PTSD, there is clearly a need for a more comprehensive approach in understanding how the whole brain responds to trauma. In other words, the imbalance of the orchestrated brain network may be clinically more relevant than specific subset connectivity changes. This also avoids risk of bias from a priori selection of specific regions or networks.

Modeling the brain as a network enables us to examine different characteristics of connections and circuitry. Graph theory examines the shape of connections between nodes (ie, brain regions) within a network. The most fundamental graph theory measure of functional connectivity is degree centrality (DC). It is defined as the sum of all edges (or connections) to a node. Kennis and colleagues^15^ recently found *decreased* DC (ie, a lower number of brain regions functionally connected to a given node) in orbitofrontal regions; right superior and inferior temporal gyrus; and left angular and superior parietal gyrus in a sample of 53 veterans with PTSD, as compared to 29 veterans without PTSD, and 26 healthy civilians. One of their limitations was that their method of analysis required strong multiple comparison correction (statistics corrected for 90 regions). While this diminishes the risk of false positives, it also increases the potential of decreasing test sensitivity, and the risk of missing true findings. This trade-off is a common challenge in whole-brain neuroimaging analysis.^16^

Our study proposes a novel analytic approach that combines graph theory^17^ and scaled subprofile modeling^18^ (SSM), which identify degree centrality and dominant group-discriminating topographical patterns, respectively. SSM is a form of principal component analysis (PCA), standardized by Eidelberg and colleagues^2,18,19^ for brain imaging analysis. SSM not only captures the most discriminative features between groups – it enables one to quantify how much an individual’s brain network resembles a pathological brain network configuration. If two distinct groups (eg, disease vs. control) are pooled, SSM can characterize the disease-related brain activity covariance pattern that differentiates the two groups if such characteristics exist.^18^ SSM can evaluate the relationships between all features analyzed (such as graph theory metrics). SSM presents the combinations (or principal components) of these measures that create the most powerfully discerning features. This covariance pattern is also associated with subject scores, indicating how greatly each brain expresses this disease-related configuration. This allows for quantifiable comparison of individual topographical profiles. This method has demonstrated utility in other neurological disorders,^20^ in early differential diagnosis,^21,22^ and in clinical trials.^23,24^ It has also exhibited high reproducibility.^25–27^

In this study, we investigated group differences of resting-state fMRI-based intrinsic connectivity in known resting state networks (RSNs) and graph theory-based measures between 11 patients with PTSD in our first cohort (PTSD-A) and 11 trauma-exposed controls (TEC). These 22 subjects were used for the combined analytic approach integrating graph theory and SSM that offers the potential to identify more useful biomarkers than either in isolation. We subsequently tested its performance in a new cohort (n = 33), comprised of 22 participants (PTSD-B) and 11 age-matched trauma-naïve healthy controls (TNC), scanned with a different MRI scanner. These 33 subjects were from an ongoing study at a different research center, therefore using different acquisition parameters. We hypothesized that a pattern able to reliably distinguish PTSD from controls could be identified by our novel approach, and could be validated with this new cohort to confirm transferability of our method.

## Methods

### Study Approval

All recruitment and testing procedures were approved by the ethics review boards of Ryerson University, the University Hospital Network (Toronto, ON), and the University of Manitoba (Winnipeg, MB). All participants provided written informed consent prior to participating. All procedures were performed in accordance with relevant guidelines and regulations.

### Participants

Clinical and demographic information, inclusion/exclusion criteria and limited imaging data (not including any resting-state fMRI) of cohort A (PTSD-A and TEC) subjects have been reported elsewhere.^28^ Twenty-two participants were recruited into one of two groups: those with a current diagnosis of PTSD (PTSD-A group; n = 11) and those who had experienced a traumatic event, but were never diagnosed with PTSD secondary to their traumatic event (TEC group*;* n = 11). Additionally, baseline scans of 33 participants (TNC n = 11, PTSD-B n = 22) were included as cohort B, from a current ongoing clinical trial and evaluated for our PTSD-related pattern. TNC subjects had not been previously exposed to trauma and had no psychiatric disorders.

### Measures

#### Toronto cohort/cohort A (PTSD-A and TEC)

Diagnosis for PTSD using the Clinician Administered PTSD Scale (CAPS) for DSM-IV stipulates endorsement of one symptom from the intrusion symptoms cluster, two from the arousal and reactivity cluster, and one from the avoidance/numbing cluster. CAPS-IV scores rate each symptom using frequency and intensity scores; for a symptom to be included toward meeting the CAPS-IV diagnostic threshold, the individual must score at least 1 on frequency and 2 on intensity. PTSD-A individuals were required to meet diagnostic criteria and have a total CAPS-IV score of 45 or greater, while TEC participants needed a CAPS-IV score of ≤30. The relevant information is summarized in Table 1.

**Table 1.** Demographics and clinical characteristics.

	Cohort A (Toronto)		Cohort B (Winnipeg)		Significance
	TEC (n = 11)	PTSD-A (n = 11)	TNC (n = 11)	PTSD-B (n = 22)	*p*
**Age**	31.45 ± 10.71	34.36 ± 13.6	28.0 ± 8.8	36.91 ± 12.65	.217
**Gender (M:F)**	3:8	5:6	3:8	8:14	.771
**Education (years)**	14.36 ± 2.25	13.91 ± 2.47	16.18 ± 1.78*	13.84 ± 2.19	.035
**BDI-II**	6.0 ± 8.3	18.9 ± 12.5			.01
**MINI MDE (present:absent)**			0:11	17:5	<.001
**Medication Status (psychotropics:none)**	2:9	5:6	n/a	12:10	0.136
**CAPS-IV**	13.5 ± 10.2	70.1 ± 14.2			<.001
**CAPS-5**			n/a	34.09 ± 9.83	

CAPS: Clinician-Administered PTSD Scale;^42^ BDI-II: Beck Depression Inventory second edition;^41^ M.I.N.I. MDE: Mini International Neuropsychiatric Interview^30^ Major Depressive Episode (past, current, or recurrent); PTSD: Posttraumatic stress disorder; TEC: trauma-exposed control; TNC: trauma-naïve control; Diff: for statistical difference between groups, either one-way ANOVA, t-test, or Chi-Square test was used. Data indicate mean ± standard deviation. Post-hoc Bonferroni revealed a significant difference in education (*) between PTSD-B versus TNC (p = 0.033).

#### Winnipeg cohort/cohort B (PTSD-B and TNC)

Individuals were evaluated for PTSD diagnosis using the CAPS for DSM-5 (CAPS-5). This updated diagnostic criteria includes a new symptom cluster and a different scoring system while maintaining continuity and compatibility with CAPS-IV assessment.^29^ Symptom severity rating is scored differently in the CAPS-5, with each symptom rating of 0–4 based on a *combination* of frequency and severity. Therefore, a lower score on the CAPS-5 versus the CAPS-IV does not necessarily reflect lower severity. Diagnosis requires at least one intrusion symptom, one avoidance symptom, two hyperarousal symptoms, and two negative cognition and mood symptoms (thus, a minimum score of 12 would be required). 18 PTSD-B participants met CAPS-5 diagnostic criteria, while the remaining 4 did not meet this threshold. However, all PTSD-B individuals exhibited more than 6 symptoms meeting a moderate (threshold) score. All PTSD-B participants had clinically significant symptoms (ie severe enough to seek treatment), with a mean CAPS-5 score of 34.09 (Table 1), ranging from 17–50. Exclusion criteria for PTSD-B participants included any neurological illness, as well as substance dependence and/or uncontrolled bipolar or psychotic disorder, as defined by the Mini International Neuropsychiatric Interview (M.I.N.I.).^30^ Co-occurring depression was also assessed using the M.I.N.I. (Table 1), but was not an exclusionary criterion.

### Resting-State fMRI Data Acquisition and Image Preprocessing

For the PTSD-A and TEC groups, anatomical and functional data were acquired on a 3-T Sigma MR System (GE Medical Systems, Milwaukee), located at Toronto Western Hospital. Anatomical scans, for co-registration of functional data, were acquired first using a fast spoiled gradient echo (FSPGR) sequence (**T**_1_-weighted sequence, 176 slices, FOV = 256 mm, slice thickness = 1 mm, 0 gap, 256 × 256 matrix, resulting in a voxel size of 1.0 × 1.0 × 1.0 mm^3^). The resting-state scan was 308 seconds long (TR: 2000 ms, TE: 30 ms, flip angle: 85°, axially oriented slices: 40, voxel size: 3.125 × 3.125 × 4.0mm, FOV: 200 mm). Participants lay still during the resting-state scan with eyes open while viewing a black fixation cross on a white screen.

For the PTSD-B and TNC groups, all subjects underwent MRI scans using a 3T Siemens/IMRIS MR System equipped with a 12 channel head coil, located at Health Sciences Centre (Winnipeg). The resting-state scan was 11 minutes (TR: 2000 ms; TE: 28 ms, flip angle: 77°; slice thickness = 4 mm; voxel size: 3.4 × 3.4 × 4.0 mm; FOV: 220 mm). Participants were instructed to keep their eyes open (no fixation point), and to let their mind wander but to not fall asleep. A high-resolution **T**_1_-weighted image was acquired using a 3D structural MPRAGE dataset (TI: 900 ms; TR: 2300 ms; TE: 3.02 ms; flip angle: 9°; slice thickness = 1 mm, for 240 slices; voxel size: 1.0 × 1.0 × 1.0 mm; FOV: 256 mm) for a scan of 5 minutes.

Standard preprocessing has been applied to the fMRI data using CONN (http://nitrc.org/projects/conn). The fMRI data were co-registered to participants’ structural T1-MRI scans, spatially normalized to template MRI (Montreal Neurological Institute space), then smoothed (FWHM = 8mm × 8mm × 8mm). Individuals’ T1 images were segmented and a gray matter probability map was constructed for masking (inclusive) the 118 different ROIs defined by Automated Anatomical Labeling (AAL^31^^)^ which additionally include left and right pons.^32^ For subcortical ROIs (ie, caudate, putamen, pallidum, thalamus and pons) a white matter probability map was additionally included for inclusive masking, since gray matter map alone does not fully delineate these subcortical regions. For denoising, linear regression was performed with confounding variables of white matter, cerebrospinal fluid, realignment, scrubbing and global signal. Then, band-pass filter was applied (0.008-0.09 Hz) and linear detrending was performed.^33^

### Intrinsic Connectivity Analysis of Known RSN

Intrinsic connectivity is a network-level measure of nodal centrality - it is defined as the root mean square of correlation coefficients between a selected voxel or region of interest (ROI) and the rest of the brain.^34^ Voxel-wise intrinsic connectivity analysis was performed using CONN. Based on the previous literature review,^9,35–37^ 32 ROIs from functional networks were selected, including nodes of the Default Mode, Frontoparietal, Salience, Dorsal Attention, Visual, Sensorimotor, Language, and Cerebellar Networks. The ROIs were taken from networks.nii image file pre-packaged in CONN software, which collected commonly used networks and areas that are defined by independent component analysis using Human Connectome Project data.^33^ The mean intrinsic connectivity was estimated for PTSD-A and TEC, then compared between the two groups using independent t-tests.

### Graph Theory Analysis

The region-to-region connectivity matrix (z-matrix) was constructed using individually gray matter-masked automated anatomical labeling (AAL^31^^)^ ROIs. The z-matrix was sorted and adjacency matrices were defined with varying sparsity (1-50%). Here we define sparsity as the number of edges that have been represented in the network. For example, at 25% threshold, the top 25% of the z-values were set to 1 and the rest were set to 0 excluding the diagonal elements. The graph was undirected, and both positive and negative connectivity have been considered.^32,38^ Graph theory metrics including characteristic path length, clustering coefficient, smallworldness, global and mean local efficiency were compared between groups.^17,39,40^ At a regional level, degree centrality was calculated by summing all edges in each node. The Brain Connectivity Toolbox (Rubinov and Sporns 2010) and in-house programs running on MATLAB 8.3.0 (Mathworks, Inc.) were used.

### Identifying a Degree Centrality Pattern Reliably Distinguishing PTSD-A from TEC

Characteristic covariance patterns that were successful in separating groups (PTSD-A vs. TEC) were identified as principal components (PCs) using SSM (http://www.fil.ion.ucl.ac.uk/spm/ext/#SSM). Stepwise linear regression was performed to select the relevant PCs, ie, a PC is included if model fit for group differentiation is significantly improved (p < 0.05).^18^ Only those PCs with >5% variance-accounted-for were considered. In other words, the combination of PCs that most significantly and reliably differentiated between PTSD-A and TEC were selected. To estimate regional contribution to overall topography, bootstrap resampling test (n = 10,000) was performed to identify significant ROIs that produce consistent region weights outside of 95% confidence interval (p *<* 0.05, two-tailed).

The topographical profile of each participant's degree distribution (ie, subject ‘scores’ reflecting the similarity of each individual's network configuration to the identified pattern) were then estimated for all groups (PTSD-A, TEC, PTSD-B, and TNC) as previously described.^18^

### Statistical Analysis

Global graph theory metrics (characteristic path length, clustering coefficient, global efficiency, mean local efficiency and smallworldness) and regional centrality (degree of each node; q < .05 corrected by false discovery rate) were compared between PTSD-A versus TEC using independent t-tests. Subject scores of the identified pattern were also compared between PTSD-A and TEC using independent t-tests. Leave-one-out cross validation was performed to test the replicability of the proposed method in estimating subject scores (ie, pattern expression) in a prospective cohort.

Pearson correlation was used to investigate the relationship between clinical symptom severity (CAPS-5 score) and identified pattern scores in PTSD-B. One-way ANOVA and post-hoc Bonferroni test were used to examine the group differences of the identified pattern scores (PTSD-A vs. TEC vs. PTSD-B vs. TNC). For all variables, normality was confirmed using Shapiro-Wilk.

## Results

### Demographics

There was no significant difference between groups with respect to age or sex (Table 1). Education differed slightly between groups (F(3,51) = 3.098, p = 0.035), with post-hoc Bonferroni test revealing a difference between PTSD-B versus TNC (p = 0.033). Beck Depression Inventory-II (BDI-II^41^^)^ scores were significantly higher in PTSD-A versus TEC (t(20) = −2.856, p = 0.01), and significantly correlated with CAPS-IV^42^ scores (r = 0.62, p = 0.002, PTSD-A and TEC pooled). Medication status was not significantly different among TEC, PTSD-A, and PTSD-B (□^2^ = 3.983, p = 0.136). No significant difference was found in BDI-II scores between cohort A patients taking anti-depressants versus patients who do not (t(9) = .834, p = 0.426).

### Intrinsic Connectivity Analysis Within Known RSNs

**Figure 1.**
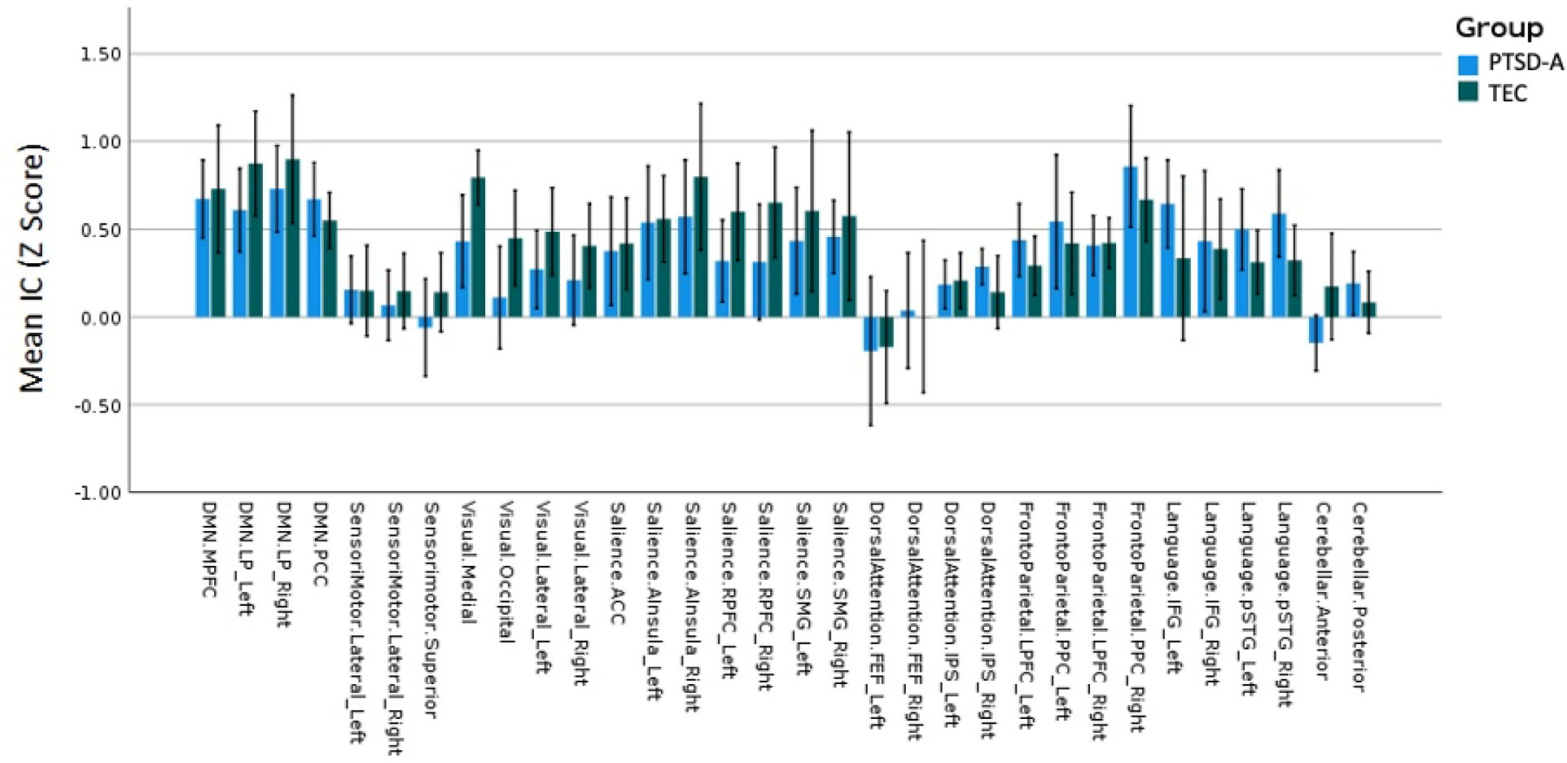
Intrinsic connectivity. Intrinsic connectivity (voxel-wise correlation to the rest of the brain) within 32 ROIs from known RSNs were compared between PTSD-A (n = 11) and TEC (n = 11). PTSD-A participants had significantly lower intrinsic connectivity than TEC (t(20) = −2.372, p = 0.028) in the Visual Medial (Visual.Medial) ROI. Data represent mean ± standard error.

Intrinsic connectivity (voxel-wise correlation to the rest of the brain) within 32 ROIs from known resting state networks were compared between PTSD-A and TEC (Figure 1). PTSD-A participants had significantly lower intrinsic connectivity than TEC (t(20) = −2.372, p = 0.028) only in the medial ROI of the visual network (MNI coordinates centred at 2, −79, 12). This finding is not statistically significant if a stricter correction for multiple comparisons is applied.

### Graph Theory Analysis

**Figure 2.**
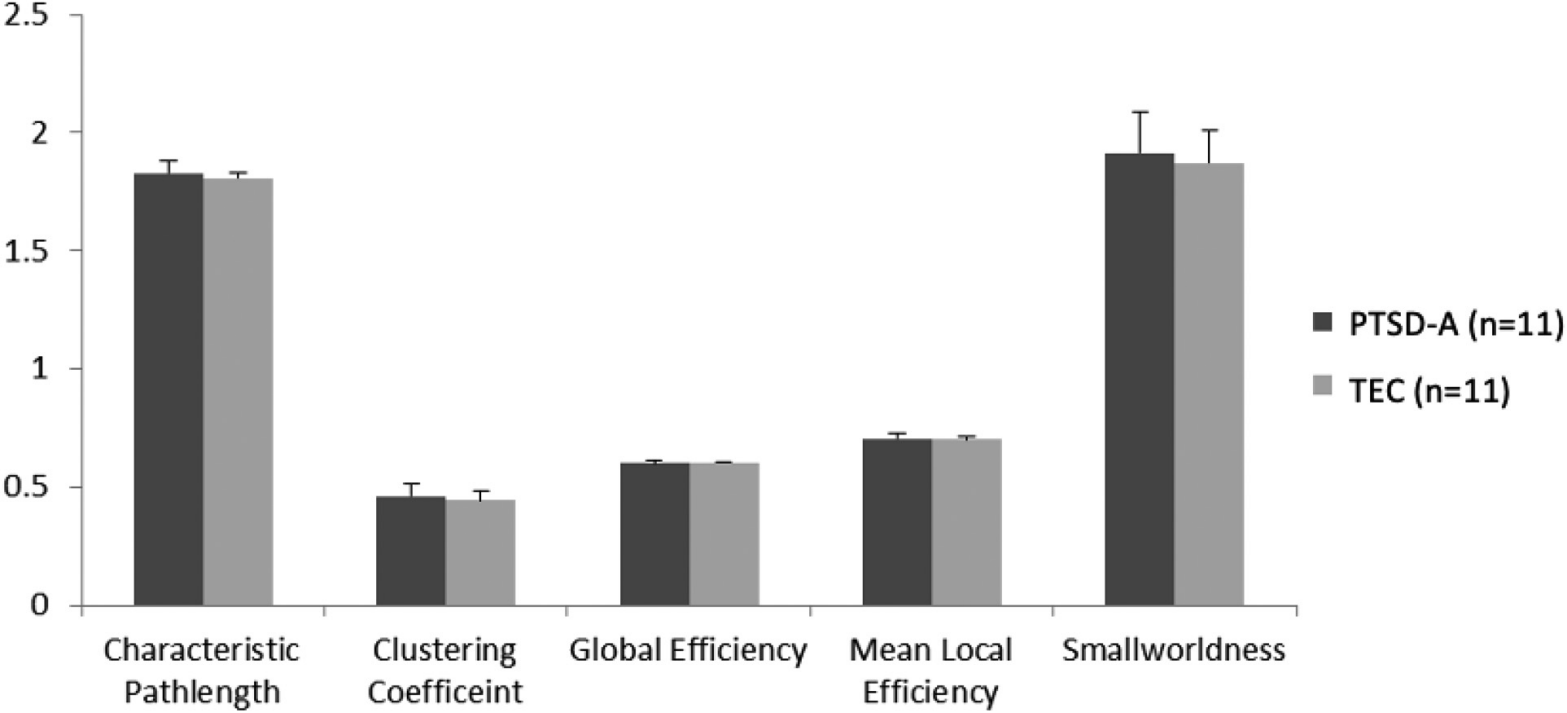
Global network metrics of graph theory analysis. No significant difference was observed between PTSD-A (n = 11) and TEC (n = 11) groups using independent t-tests for characteristic path length, clustering coefficient, global efficiency, mean local efficiency and smallworldness (p > .2). Data represent mean ± standard deviation.

Binarized undirected graphs were constructed from each individual's resting-state fMRI. Individuals’ graphs began to be fully connected at the range of threshold 17–24% sparsity, thus the graphs were analyzed at the minimal sparsity that ensured full connectedness in all individuals (24%). The minimum sparsity that produced a fully connected graph was not significantly different between PTSD-A and TEC (t(30) = .778, p = 0.443). Unlike previous resting-state fMRI studies in PTSD,^12^ we did not observe any significant changes in global graph theory metrics, ie, clustering coefficient (t(20) = .950, p = 0.354), characteristic path length (t(20) = 1.237, p = 0.230), global efficiency (t(20) = -1.232, p = 0.232), mean local efficiency (t(20) = 0.824, p = 0.420) or smallworldness (t(20) = 0.656, p = 0.519) (Figure 2). At a regional level, degree centrality (DC) was calculated by summing all edges in each node. No significant difference in DC was observed at nodal level cf^43,44^ between PTSD-A and TEC after correction for multiple comparisons using false discovery rate (FDR) (q(FDR) > .14). However, trends (ie, p < 0.05, q(FDR) > 0.05, ie, significant at uncorrected level) of lower DC in PTSD-A were observed in the left superior occipital cortex and cerebellar regions (Supplementary Table S1).

### Spatial Pattern Analysis of Degree Centrality

**Figure 3.**
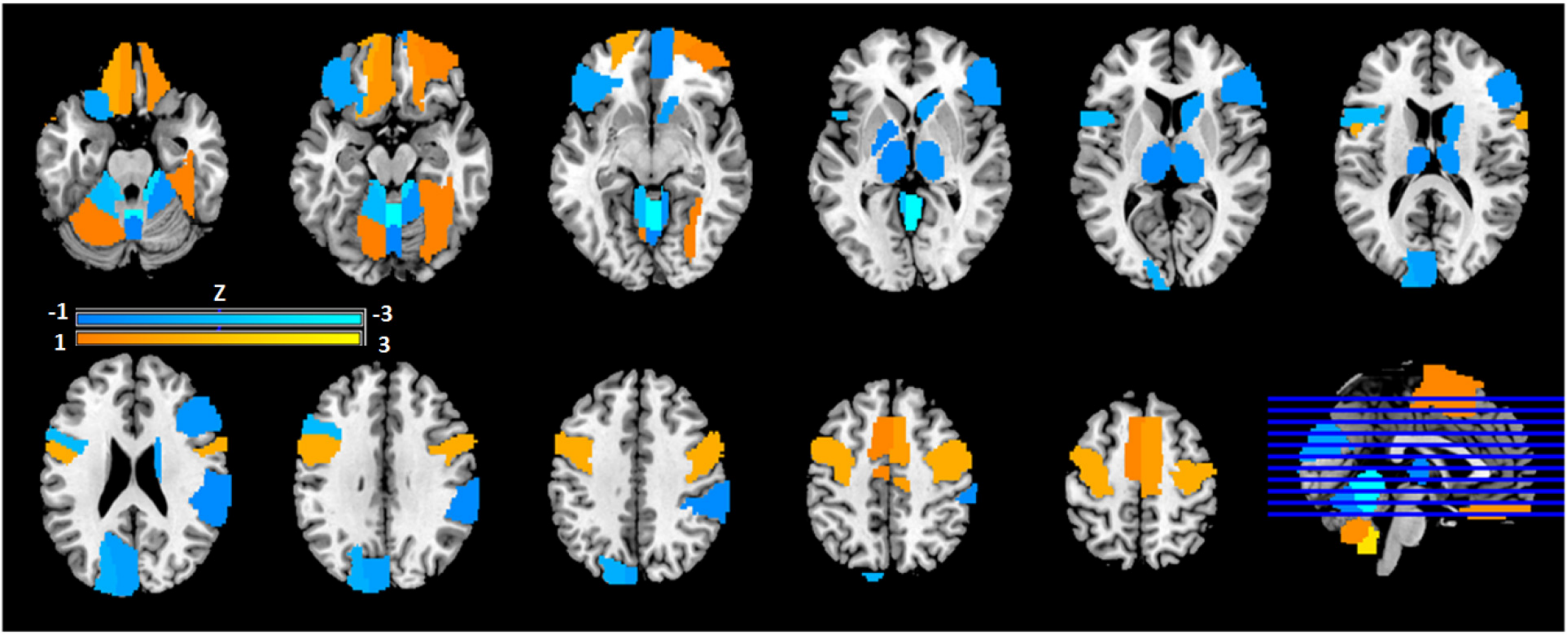
Topographical distribution of PTSD-related functional connectivity pattern. SSM analysis^18^ was conducted to the degree centrality matrix of subject × ROIs to identify principal components. Stepwise regression analysis was performed to define a group discriminating pattern resulted in a linear combination of fourth and sixth principal components, accounting for 13.8% of total covariance. Region weights of the resulting pattern were z-scored to the mean of the whole-brain (refer to Supplementary Table S2). Relevant centrality region weights (|Z|>1) are visualized. Warm colors (orange/yellow) represent regions of heightened degree centrality; cool colors (blue) represent reduced degree. centrality.

**Figure 4.**
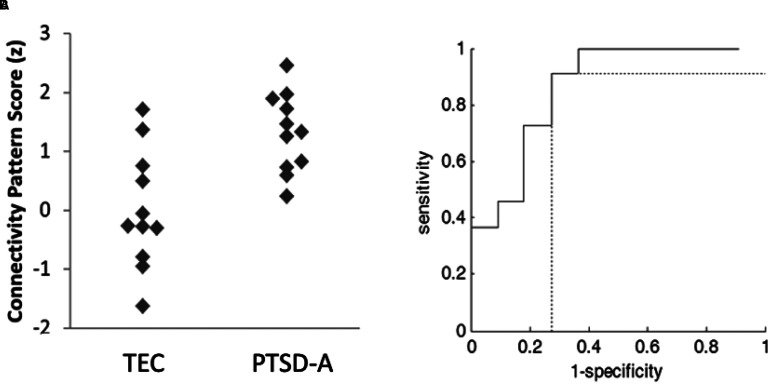
Training set PTSD-related functional connectivity pattern scores. Topographical profiles (ie, subject scores) of each subject were computed as described elsewhere.^18^ The participant scores were z-scored to the mean of TEC group. **A.** PTSD-A patients (n = 11) showed significantly higher pattern scores than the TEC (n = 11) group (t(20) = 3.61, *p <* .002). **B.** Good area under the curve (AUC = .86) was achieved by receiver-operating-curve analysis with excellent sensitivity ( = .91) and moderate specificity ( = .73).

**Figure 5.**
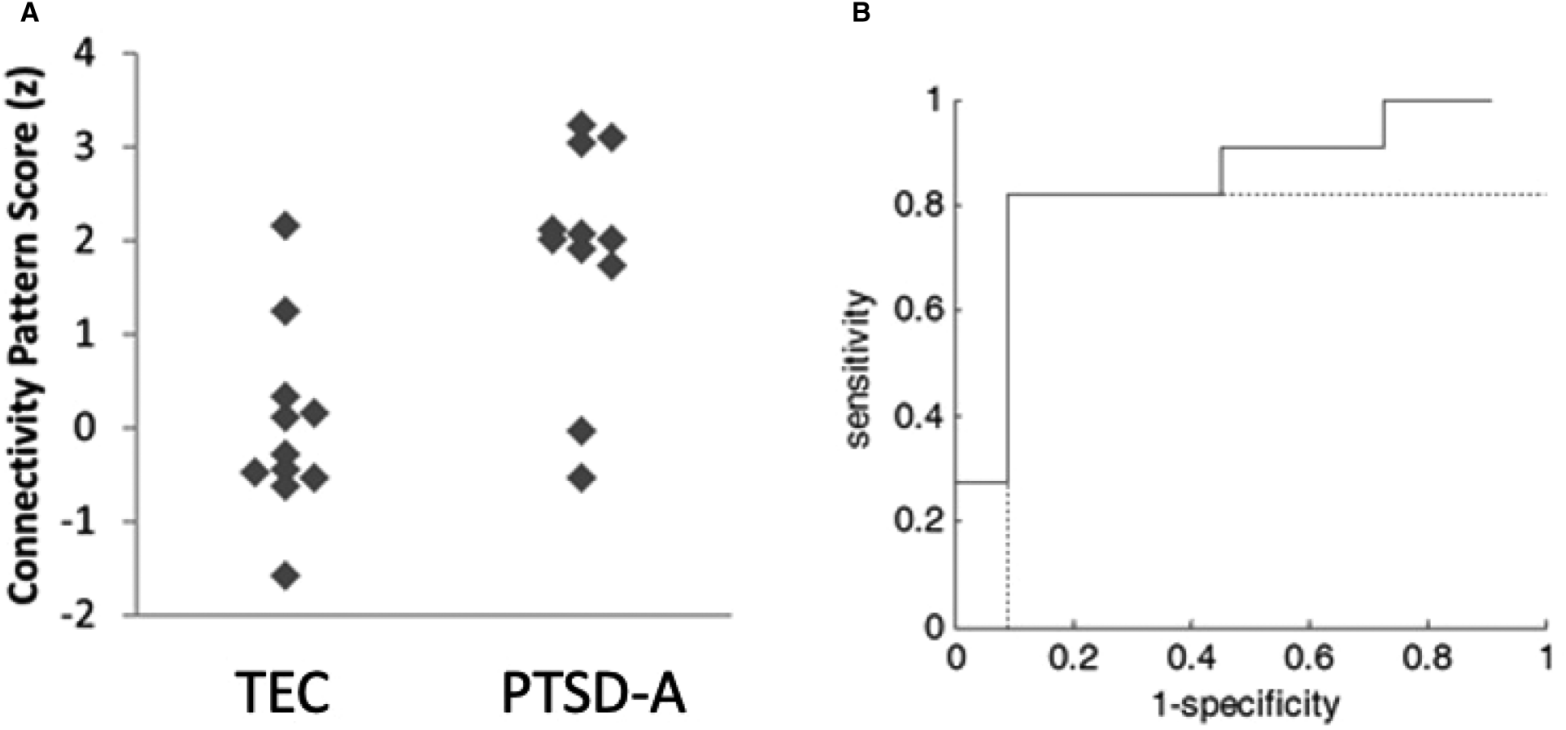
Training set leave-one-out cross validation of PTSD-related functional connectivity pattern. The pattern scores were calculated by leave-one-out procedures, then the participant scores were z-scored to the mean of TEC group. A. Patients with PTSD (n = 11) showed significantly higher pattern scores than the TEC (n = 11) group (t(20) = 3.98, p < 0.001). B. Good area under the curve (AUC = .84) was achieved by receiver-operating-curve analysis with preserved sensitivity ( = .82) and specificity ( = .91).

The SSM was used to identify the characteristic spatial distribution of a pattern of degree centralities that differentiated PTSD-A versus TEC (Figure 3). Stepwise linear regression with principal components (PC) was performed to construct the group differentiating pattern. The resulting spatial pattern was linear combination of fourth and sixth PCs accounting 13.8% of total covariance, and pattern expression was significantly different between PTSD-A and TEC (t(20) = 3.61, p *<* .002; Figure 4A). Receiver-operating curve analysis revealed excellent sensitivity ( = .91) and moderate specificity ( = .73) (Figure 4B). This PTSD-related pattern is characterized by increased DC in inferior orbitofrontal regions, precentral gyri, supplementary motor areas, right fusiform, left middle temporal pole, and inferior cerebellar regions (Figure 3; Supplementary Table S2). Decreased DC was observed in the bilateral thalamus, left pallidum, right caudate, superior orbitofrontal regions, occipital regions, left cuneus and superior cerebellar regions (Figure 3; Supplementary Table S2). Bootstrap resampling tests revealed two ROIs consistently below or above 95% CI (left pallidum and right cerebellar lobule 9, respectively). Leave-one-out cross validation analysis revealed that group separation according to the pattern score is highly replicable (t(20) = 3.98, p < .001; Figure 5A) with preserved sensitivity ( = .82) and specificity ( = .91) (Figure 5B).

### Transferability of Identified Pattern

**Figure 6.**
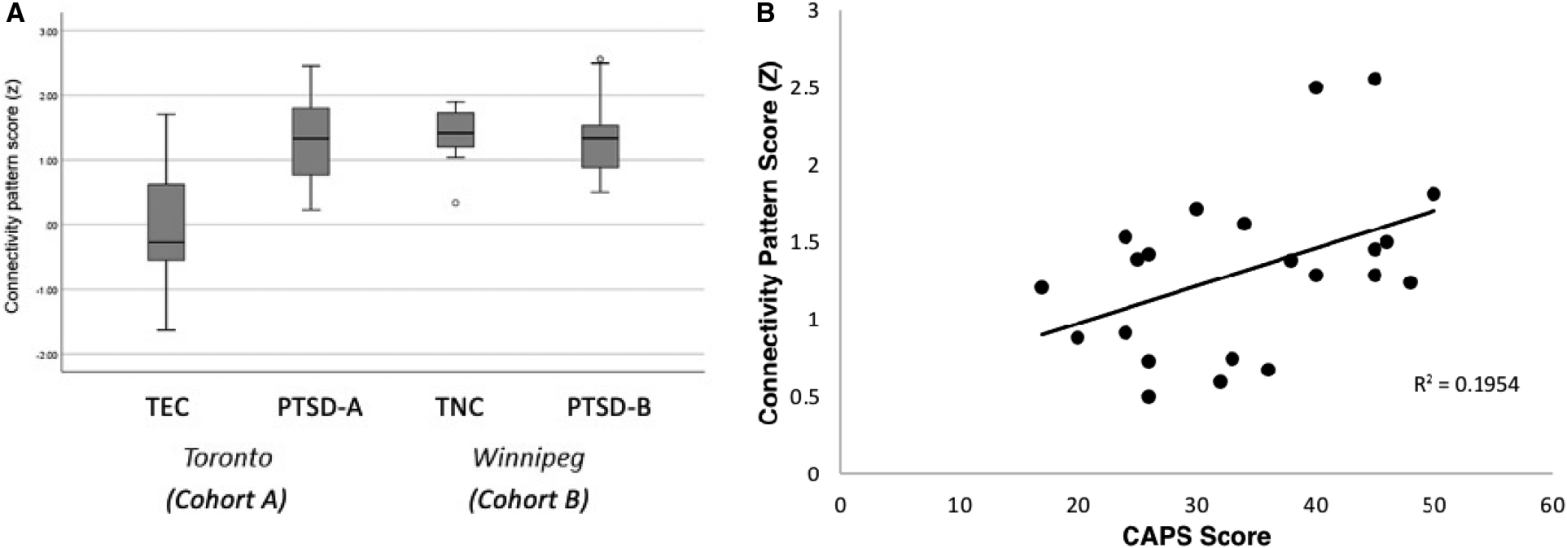
A. Group differences of PTSD-related functional connectivity pattern scores. The pattern scores were z-scored to the mean of TEC group. One-way ANOVA (F(3,51) = 11.676, *p* < 0.00001) with post hoc Bonferroni test revealed TEC (n = 11) subject scores to be significantly lower than PTSD-A (n = 11), PTSD-B (n = 22), and TNC (n = 11) (*p* < .0005). There were no significant differences between the two PTSD groups, or within the two testing set groups. B. Clinical correlates with PTSD-related connectivity pattern scores in PTSD-B**.** CAPS-5 scores in PTSD-B participants (n = 22) showed a positive correlation (R = 0.442, p = 0.039) with subject scores for the PTSD-related connectivity pattern (z-scored here to the mean of TEC group), using Pearson’s correlation test.

Topographical profiles (subject scores) of the pattern were calculated in all subject groups (PTSD-A, TEC, PTSD-B, and TNC). A one-way ANOVA showed a significant effect of group in the pattern scores across the 4 groups (F(3,51) = 11.676, p < 0.00001; Figure 6A). Post-hoc Bonferroni tests revealed that the TEC group's pattern scores were significantly lower than all other groups (p < 0.0005); however, both PTSD groups and TNC were not significantly different from each other (p = 1.000). PTSD-B patients’ CAPS-5 scores showed a positive correlation with their PTSD-related connectivity pattern scores (r = 0.442, p = 0.039; Figure 6B). In other words, higher symptom severity was positively correlated with higher expression of the PTSD-related pattern.

The PTSD-B group was subdivided into two groups: either presence or absence of major depressive episode diagnoses as measured by the M.I.N.I.^30^ (diagnoses of past, current, or recurrent major depressive episodes). A t-test revealed no significant difference between the two groups with respect to their PTSD-related pattern expression scores (t(20) = .047, p = 0.830).

## Discussion

While the conventional intrinsic connectivity analyses did not reveal any significant group differences between PTSD-A and TEC, our novel approach led to the discovery of a spatially covarying pattern of degree centrality that differentiated PTSD-A patients from the TEC sample. As no correlation was observed between SSM-identified pattern scores and BDI-II scores, it is unlikely our main finding can be explained by comorbid depression or anti-depressant treatment alone.

For cohort A, maximizing the differences between the two groups (PTSD-A vs. TEC) was important for our SSM prediction model to identify the group differentiating pattern as principal components with non-negligible covariance (>5%), and thus stricter diagnostic critera without overlap between the two groups was applied (CAPS-IV ≥ 45 vs. CAPS-IV ≤ 30). For cohort B, however, our purposes were to examine if the proposed PTSD-related function connectivity pattern has clinical relevance (ie, correlation with symptom severity in PTSD-B) and to validate if the pattern expression was developed as a response to trauma exposure. Thus, the PTSD-B group included wider range of PTSD symptom severity and 4 of 22 patients did not meet CAPS-5 diagnostic threshold. Interestingly, the non-difference of the pattern expression in TNC versus PTSD-B complicates the interpretation and singles out TEC from the rest of the groups (PTSD-A, PTSD-B, and TNC), which may suggest that the identified PTSD-related functional connectivity pattern expression may, in fact, be related with compensatory mechanisms (see below for further discussion).

Bootstrap resampling tests for region weights revealed that the identified pattern cannot easily be localized to a few key ROIs (ie, only 2 out of 118 region weights were consistently outside of 95% CI), thus further emphasizing the use of whole-brain topology approach such as SSM-PCA in interpreting PTSD-related brain networks.

Here it should be noted that our graph theory approach incorporates both positive and negative connectivity indiscriminately^38^ and edges are undirected. This approach considers inhibitory connections as important as facilitative connections. Indeed, when only positive connections (positive z-value in the region-to-region connectivity matrix) are considered, no group differentiating PC was identified (data not shown).

Our spatial pattern is comprised of many regions consistently implicated in PTSD connectivity studies, but not traditionally regarded as the most salient components in PTSD pathophysiology.^9,15,45^ While TNC subject profiles were not significantly different from either PTSD groups (Figure 6A), TEC demonstrated significantly lower expression of the pattern compared to all other groups (PTSD-A, PTSD-B, and TNC). In contrast to what we expected, regions such as the amygdala, hippocampus, insula, and cingulate cortices were not critically involved with our network configuration. One interpretation is that an inverse of our degree centrality pattern, expressed more highly in TEC than either PTSD or TNC, might represent compensatory mechanisms secondary to the trauma exposure and consequent development of PTSD. If so, it is possible that regions traditionally found most important in PTSD pathophysiology would not necessarily be most significant here. Rather, subcortical structures, occipital and cerebellar cortices, which have shown *modulatory* functions on limbic structures and in sensory and emotional processing,^46,47^ have been deemed salient in our pattern. This could explain the dichotomy of our TEC subjects’ scores from TNC and PTSD groups.

TEC subjects had significantly higher intrinsic connectivity in the visual medial network. The lack of significant difference in any nodes from the triple network model is actually unsurprising, in comparison with previous studies. For example, Abdallah and colleagues^48^ found high salience network intrinsic connectivity (referred to as global brain connectivity in the paper) as compared to asymptomatic combat controls. However, this was only during task-based fMRI; resting state investigations found salience network intrinsic connectivity did not differ between groups. They also did not find any difference in CEN connectivity between groups at rest.

### Limitations

Our analysis is limited by the regions of interest defined. We know from previous research that structures such as the hippocampus and amygdala are not homogeneous, though they are denoted so by AAL^31^ parcellations. They have discrete subregions serving separable functional roles, which have displayed differential connectivity between PTSD and TEC individuals in resting state fMRI investigations.^49,50^ Future research employing graph theory and SSM may reveal a more sensitive distinguishing pattern by using regions that are defined by both anatomical and functional properties.

All graph theory metrics were calculated under the assumption that the pattern of whole-brain connectivity is similar in PTSD-A and TEC. This is inevitable as comparing metrics from graphs with different sparsity thresholds is unjustified^17^ and it is not possible to identify the “truly connected edges” from the region-by-region correlation map derived by resting-state fMRI. It has previously been demonstrated that using different thresholding strategies for graph construction (eg, using the minimum threshold that results in fully connected graphs vs. averaging the metrics over multiple graphs that are defined at varying range of thresholds that are arbitrarily defined) can cause large variability in graph theory-based metrics,^17,51^ which is not limited to human brain imaging studies. These methodological differences may have caused some discrepancy in our results compared to the previous studies where changes in global graph theory metrics were identified.^12^

The relatively small sample size may have prevented us from identifying previously reported significant changes in global graph theory metrics, ie, clustering coefficient, characteristic path length, global efficiency, mean local efficiency and smallworldness.^12^ Nevertheless, our novel approach combining graph theory and SSM was sensitive enough to detect group differences between PTSD and TEC. Most importantly, the reliability of the proposed method has been validated rigorously using leave-one-out cross-validation procedure.

## Conclusion

The combined approach of graph theory analysis and scaled subprofile modeling has been used to successfully determine neurological disorders and assist diagnoses. Here, we present its utility in characterizing psychiatric disorders, such as PTSD. Future exploration of this approach, including a larger sample size, may pave the way toward development of neuroimaging biomarkers of psychiatric symptoms and disorders.

## Supplemental Material

sj-docx-1-css-10.1177_24705470221092428 - Supplemental material for Novel Analysis Identifying Functional Connectivity Patterns Associated with Posttraumatic Stress Disorder Click here for additional data file. Supplemental material, sj-docx-1-css-10.1177_24705470221092428 for Novel Analysis Identifying Functional Connectivity Patterns Associated with Posttraumatic Stress Disorder by Natalie Wright,, Ronak Patel,, Sarah J. Chaulk,, Gillian Alcolado,, David Podnar,, Natalie Mota,, Candice M. Monson,, Todd A. Girard, and Ji Hyun Ko, in Chronic Stress

## Abbreviations:

AAL: Automated anatomical labeling
BDI-II: Beck Depression Inventory II
CAPS: Clinician Administered PTSD Scale
CEN: Central executive network
DMN: Default mode network
DC: Degree centrality
DSM: Diagnostic and Statistical Manual of Mental Disorders
fMRI: Functional magnetic resonance imaging
PTSD: Posttraumatic stress disorder
PCA: Principal component analysis
ROI: Region of interest
SN: Salience network
SSM: Scaled subprofile modeling
TEC: Trauma-exposed controls
TNC.: Trauma-naïve controls
