# Supplementary material for: Novel Analysis Identifying Functional Connectivity Patterns Associated with Posttraumatic Stress Disorder
Source: Chronic Stress (Thousand Oaks). 2022 Apr 18;6:24705470221092428. doi: 10.1177/24705470221092428 (PMC9019376; doi:10.1177/24705470221092428)
Supplement: sj-docx-1-css-10.1177_24705470221092428 - Supplemental material for Novel Analysis Identifying Functional Connectivity Patterns Associated with Posttraumatic Stress Disorder [file sj-docx-1-css-10.1177_24705470221092428.docx]

**SUPPLEMENTARY MATERIAL**

**Supplementary Table 1**. Group differences in functional connectivity (degree) at minimum sparsity of graph (24%).

|  | PTSD (n=11) | | TEC (n=11) | | Statistics | | |
| --- | --- | --- | --- | --- | --- | --- | --- |
|  | Mean | STD | Mean | STD | t | p | q (FDR) |
| Right Cerebellum Lobule 9 | 28.4 | 10.6 | 19.7 | 7.5 | 2.2 | 0.039 | 0.912 |
| Left Superior Occipital | 25.6 | 8.6 | 34.6 | 9.5 | -2.3 | 0.030 | 0.912 |
| Left Cerebellum Lobule 4/5 | 25.5 | 8.2 | 35.5 | 6.4 | -3.2 | 0.005 | 0.266 |
| Vermis 4/5 | 18.7 | 7.0 | 31.7 | 9.0 | -3.8 | 0.001 | 0.142 |

**Supplementary Table 2.** Functional connectivity spatial pattern characterizing PTSD.

| **Region** | **Region Weights** |
| --- | --- |
| *Increased FC*^1^ |  |
| **Left Precentral Gyrus** | **1.7213** |
| **Right Precentral Gyrus** | **1.7329** |
| **Left Superior Frontal Gyrus (Orbital)** | **1.6965** |
| **Right Superior Frontal Gyrus (Orbital)** | **1.2621** |
| **Right Middle Frontal Gyrus (Orbital)** | **1.0865** |
| **Left Supplementary Motor Area** | **1.1212** |
| **Right Supplementary Motor Area** | **1.5069** |
| **Left gyrus rectus** | **1.3692** |
| **Right Fusiform Gyrus** | **1.061** |
| **Left Temporal Pole (Middle)** | **1.2501** |
| **Left Cerebellum 6** | **1.0176** |
| **Left Cerebellum 8** | **1.4037** |
| **Left Cerebellum 9** | **2.5772** |
| **Right Cerebellum 9** | **2.0699** |
| **Vermis 8** | **1.2711** |
| **Vermis 9** | **1.2645** |
| *Decreased FC*^1^ |  |
| **Left Inferior Frontal Gyrus (Opercular)** | **-1.9462** |
| **Right Inferior Frontal Gyrus (Triangular)** | **-1.3663** |
| **Left Inferior Frontal Gyrus (Orbital)** | **-1.5956** |
| **Right Medial Orbitofrontal Gyrus** | **-1.2303** |
| **Left Cuneus** | **-1.5139** |
| **Left Superior Occipital Gyrus** | **-1.7732** |
| **Right Inferior Occipital Gyrus** | **-1.0028** |
| **Right Supramarginal Gyrus** | **-1.2029** |
| **Right Caudate Nucleus** | **-1.4961** |
| **Left Pallidum** | **-1.1744** |
| **Left Thalamus** | **-1.176** |
| **Right Thalamus** | **-1.3533** |
| **Left Cerebellum 3** | **-2.1556** |
| **Right Cerebellum 3** | **-2.4182** |
| **Left Cerebellum 4,5** | **-1.9303** |
| **Right Cerebellum 4,5** | **-1.2989** |
| **Left Cerebellum 10** | **-1.3828** |
| **Vermis 4,5** | **-3.3463** |
| **Vermis 6** | **-1.1121** |

^1^Only the regions with region weight (|Z|>1) are considered.
